# Elimination of Aflatoxins B1 and B2 in White and Red Wines by Bentonite Fining. Efficiency and Impact on Wine Quality

**DOI:** 10.3390/foods9121789

**Published:** 2020-12-02

**Authors:** Fernanda Cosme, António Inês, Beatriz Ferreira, Davide Silva, Luís Filipe-Ribeiro, Luís Abrunhosa, Fernando M. Nunes

**Affiliations:** 1 CQ-VR, Chemistry Research Centre, Food and Wine Chemistry Lab., School of Life Sciences and Environment, University of Trás-os-Montes and Alto Douro, 5000-801 Vila Real, Portugal; aines@utad.pt (A.I.); bia_ferreira93@hotmail.com (B.F.); davidesilva@outlook.pt (D.S.); fmota@utad.pt (L.F.-R.); fnunes@utad.pt (F.M.N.); 2 Department of Biology and Environment, School of Life Sciences and Environment, University of Trás-os-Montes and Alto Douro, 5000-801 Vila Real, Portugal; 3 CEB-Centre of Biological Engineering, Campus de Gualtar, University of Minho, 4710-057 Braga, Portugal; d3024@deb.uminho.pt; 4 Chemistry Department, School of Life Sciences and Environment, University of Trás-os-Montes and Alto Douro, 5000-801 Vila Real, Portugal

**Keywords:** wine, aflatoxins, bentonite, wine safety, wine quality, chromatic characteristics

## Abstract

Aflatoxins B1 and B2 are two highly toxic mycotoxins that have been sometimes found in wines. Currently, no technological solution is available to reduce or eliminate aflatoxins from wines when they are present. Therefore, this work aims to study the efficiency of already approved wine fining agents like activated carbon, potassium caseinate, chitosan, and bentonite for aflatoxins B1 and B2 removal from white and red wines. It was observed that the fining agents’ efficiency in removing aflatoxins was dependent on the wine matrix, being higher in white than in red wine. Bentonite was the most efficient fining agent, removing both aflatoxins (10 μg/L total) from the white wine and 100% of aflatoxin B1 and 82% of aflatoxin B2 from red wine. The impact of bentonite on white wine chromatic characteristics was low (color difference, ΔE* = 1.35). For red wine, bentonite addition caused a higher impact on wine’ chromatic characteristics (ΔE* = 4.80) due to the decrease in total anthocyanins, although this decrease was only 1.5 points of color intensity. Considering the high efficiency of bentonite in aflatoxins B1 and B2 removal and despite the impact on red wine color, bentonite is a very good technological solution for aflatoxin removal in white and red wines.

## 1. Introduction

Mycotoxins are secondary metabolites produced by some fungi that develop in crops, including grapes, under certain environmental circumstances. Among them, aflatoxins are a group of highly toxic secondary metabolites produced by fungi belonging to the genus *Aspergillus*, mainly *Aspergillus flavus* and *Aspergillus parasiticus* [1,2,3] and to a lesser extent by *Aspergillus nomius* [4]. There are about 20 types of aflatoxins; however, despite their diversity, the main aflatoxins found in foods are aflatoxin B1 (AFB1), aflatoxin B2 (AFB2), aflatoxin G1 (AFG1), aflatoxin G2 (AFG2), aflatoxin M1, and aflatoxin M2. Their chemical structure is very similar, being classified as furanocoumarins because they have a coumarin nucleus associated with a furan and lactone ring. AFB1 is the predominant form and the most toxic too [5]. It is one of the most potent genotoxic agents and a strong hepatocarcinogen, often referred to as the most potent naturally occurring carcinogen [6,7,8]. It also has an immunosuppressant and nutritional interference effect [3] and mutagenic and teratogenicity [9]. Therefore, it is classified as a Group 1 human carcinogen by the International Agency for Research on Cancer (IARC), a body of the World Health Organization (WHO) [10].

The presence of aflatoxins in wines has been sometimes documented in recent years [11,12,13]. Di Stefano et al. [13] studied aflatoxins’ occurrence in 30 sweet wines from five winemaking regions in Sicilia, Italy. AFB1 was detected in 23% of the analyzed wines, but 21%, 40%, and 37% of the wines also contained AFG1, AFB2, and AFG2, respectively. The average concentration detected were of 0.025, 0.043, 0.015, and 0.027 μg/L for AFB1, AFG1, AFB2, and AFG2, respectively. In a different study, 24 red wines produced in different Spain regions were analyzed by Pérez-Ortega et al. [11], with AFB2 being the most frequently detected aflatoxin. Contaminated wine samples reached 87.5% of the analyzed ones, and the AFB2 levels ranged from 1.25 to 25.73 μg/L. Nistor et al. [14] detected in Romanian wines the highest concentrations for AFB1 and AFG1 (between 26.68 and 33.68 µg/kg). Aflatoxins and aflatoxins-producing strains have also been detected in grapes and grape must in North African countries—Lebanon and Tunisia [15,16]. Forty percent of the grape musts from twenty-seven vineyards in Lebanon contained AFB1 at levels below 0.46 μg/L [15]. In Tunisian vineyards, *Aspergillus flavus* represented 23% of the grape mycotoxin-producing fungi, producing AFB1 between 21 and 54 μg/g of culture medium [16]. The climate changes foreseen in the near future can considerably change this scenario, making mycotoxigenic fungi suffer geographical migrations towards the north. According to some authors, aflatoxin will supersede ochratoxin A (OTA) as the major mycotoxin in northern foods because temperatures will become more suitable for the thermotolerant aspergilli that produce aflatoxins [17,18]. In addition, in *A. parasiticus*, the proportion of aflatoxins B production compared to aflatoxins G production is greater at higher temperatures [19]. 

In 2018, the world wine consumption was 246 × 10^6^ hL [20], partly fostered for the health benefits associated with its moderate consumption [21]. However, increasing public concern about the potential health risks posed by mycotoxin residues in the human diet has increased the concern on wine quality and safety. Contrarily to OTA, whose levels in wines are regulated since 2006 with a maximum residue limit (MRL) of 2 μg/kg [22], there is no European Union (EU) legislation imposed for aflatoxins in wine. Nevertheless, the European Commission Regulation (EU) No 165/2010 set MRL for aflatoxins in several foodstuffs: 2–12 µg/kg for AFB1, and 4–15 µg/kg for the sum of four main aflatoxins (AFB1, B2, G1, and G2), depending on the type of commodity [23]. Therefore, it is crucial to develop technological solutions to reduce/eliminate the levels of mycotoxins in wine for preventing the entry of these contaminants into the food chain. In addition, considering the scenario of an increased incidence due to climate changes and the proven toxicity of aflatoxins, a low but continued dietary exposure can be a risk to consumers, so the mycotoxins’ levels in foods should be as low as reasonably as possible, ideally absent. Therefore, this work aimed to study, for the first time, fining strategies to eliminate aflatoxins (AFB1 and AFB2) in white and red wine and to evaluate their impact on wine phenolic composition and chromatic characteristics.

## 2. Materials and Methods 

### 2.1. Wine Samples

A white wine from the Vinho Verde region (vintage 2014) and a red wine from the Douro Valley (vintage 2014) was used with the characteristics shown in Table 1. Enological parameters were analyzed using a Fourier transform infrared spectroscopy (FTIR) Bacchus Micro (Microderm, France). Analyses were performed in duplicate.

### 2.2. Fining Experiments

White and red wine samples were spiked with AFB1 and AFB2 at a final concentration of 10 μg/L before the fining experiments (Sigma-Aldrich A6636 and A9887, respectively). This concentration was chosen based on the wine contamination data determined by Pérez-Ortega et al. [11] to simulate excessive contamination (worst scenario). The fining agents used were: activated carbon (100 g/hL) and chitosan (10 g/hL), using the maximum limits allowed by the International Organization of Vine and Wine (OIV) [24]; potassium caseinate (80 g/hL) and bentonite (120 g/hL), using the maximum dosage recommended by the manufacturer, since there are no official limits for their application in wines. The fining experiments were conducted in 250 mL graduated cylinders by mixing the wines with the adsorbents and by allowing the mixture to remain in contact for 7 days at 20 °C, simulating the standard enological practices. Red and white wines without any added fining agent were used as controls. All the experiments were performed in duplicate.

### 2.3. Aflatoxins Analysis in White and Red Wine

After the wine treatments with the different fining agents, the wines were centrifuged at 4000 rpm for 15 min. Then, 2 mL of the supernatant was collected and added to an equal volume of acetonitrile/methanol/acetic acid (78:20:2, *v*/*v*/*v*), after a strong vortex agitation, they were left overnight in the dark. The extracts obtained were vortexed and filtered through a nylon syringe filter with 0.45 μm of porosity and stored at 4 °C and analyzed by high performance liquid chromatography (HPLC) with fluorescence detection. The HPLC system included a Varian Prostar 210 pump, a Varian Prostar 410 autosampler, a photochemical post-column derivatization reactor (Aura Industries, San Diego, CA, USA), a Jasco FP-920 fluorescence detector, and a Jones Chromatography 7971 column heater set at 35 °C. The chromatographic separation was performed on a C18 reversed-phase YMC-Pack ODS-AQ analytical column (250 × 4.6 mm I.D. 5 mm), fitted with a pre-column with the same stationary phase. The samples were eluted at a 1 mL/min flow rate for 20 min with a mobile phase consisting of water/acetonitrile/methanol (3:3:1, *v*/*v*/*v*). The injection volume was 30 μL and parameters for detection: λex = 365 nm, λem = 435 nm, and gain = 1000. The retention time of AFB1 and AFB2 was approximately 15.6 and 13.4 min, respectively. The concentration of AFB1 and AFB2 in samples was determined by comparing peak areas with the calibration curve made with an analytical standard (46304-U, Sigma-Aldrich, Algés, Portugal). A stock solution 100x diluted was first prepared in the mobile phase. Then, a seven standard calibration curve with concentrations between 0.005–5 μg/mL for AFB1 and 0.0015–1.5 ug/mL for AFB2 was prepared also in the mobile phase. The limit of detection and quantification (LOD and LOQ, respectively) were calculated with a signal-to-noise ratio of 3:1 and 10:1, respectively. LOD was 0.015 and 0.019 μg/L for AFB1 and AFB2, respectively, while LOQ was 0.05 and 0.06 μg/L. All analyses were performed in duplicate.

### 2.4. Quantification of Non-Flavonoids, Flavonoids, and Total Phenols

The wine’s phenolic compounds were determined using the absorbance at 280 nm before and after precipitation of the flavonoid phenols through reaction with formaldehyde, according to Kramling and Singleton [25] and Ribéreau-Gayon et al. [26]. Using these methods, flavonoids, non-flavonoids, and total phenolic compounds were quantified in the wines. The results were expressed as gallic acid equivalents through calibration curves with standard gallic acid. All analyses were performed in duplicate.

### 2.5. Color, Total and Colored Anthocyanins, Polymeric and Total Pigments, and Chromatic Characteristics

White wine color (A_420 nm_) and the red wine color intensity (A_420 nm_ + A_520 nm_ + A_620 nm_) and hue (A_420 nm_/A_520 nm_) were quantified as described in the OIV methods [27]. The concentration of total anthocyanins from red wine was determined by the SO_2_ bleaching procedure using the method described by Ribéreau-Gayon and Stronestreet [28], and the colored anthocyanins, total and polymeric pigments from red wine were determined according to Somers and Evans [29]. For the chromatic characteristics of white and red wine, the absorption spectra of wine samples were scanned from 380 to 780 nm using a 1 cm path length quartz cell, and the wines’ chromatic characteristics L* (lightness), a* (redness), and b* (yellowness) coordinates were calculated using the International Commission on Illumination (CIE) method using the L*, a*, b* coordinates according to OIV [27]. The chroma (C* = [(a*)^2^ + (b*)^2^]^1/2^]) and hue-angle (h^o^ = tang^−1^(b*/a*)) values were also determined. To distinguish the color more accurately, the color difference was calculated using the following equation: ΔE* = [(ΔL*)^2^ + (Δa*)^2^ + (Δb*)^2^]^1/2^. This allows reliable quantification of the overall color difference in a sample compared to a control sample (untreated wine). Analyses were performed in duplicate.

### 2.6. High-Performance Liquid Chromatography (HPLC) Analysis of Anthocyanins, Catechin, and Phenolic Acids

Analyses were carried out with an Ultimate 3000 Dionex HPLC system equipped with a PDA-100 photodiode array detector (Dionex, Sunnyvale, CA, USA) and an Ultimate 3000 Dionex pump. The separation was performed on a C18 column (250 mm × 4.6 mm, 5 μm particle size, ACE, Aberdeen, Scotland) with a 1 mL/min flow rate at 35 °C. The injection volume was 50 μL, and the detection was performed in the wavelength range of 200 to 650 nm. The analysis was carried out using 5% of aqueous formic acid (A) and methanol (B), and the gradient was as follows: 5% B from zero to 5 min followed by a linear gradient up to 65% B until 65 min and from 65 to 67 min down to 5% B [30,31,32].

### 2.7. Statistical Analysis

The data are presented as means ± standard deviation. Analysis of variance (ANOVA) and a post-hoc test, Tukey honestly significant difference (HSD, 5% level) test was applied to physicochemical data to determine significant differences between the fining treatments. The model was statistically significant when *p* < 0.05 using the Statistica 7 software (Statsoft., Tulsa, OK, USA).

## 3. Results and Discussion

### 3.1. Aflatoxins Removal from Wines Using Different Fining Agents

There is a lack of knowledge about the most efficient fining agent for the removal of aflatoxins in the wine matrix as well in other foods, therefore as a first approach to this problem, we explored the use of four fining agents with a different interaction mechanism: electrostatic (bentonite, potassium caseinate, and chitosan) and physical interaction (activated carbon). Chitosan is a positively charged fining agent, while bentonite is a negatively charged one. Potassium caseinate is an amphoteric charged material. These materials were applied to wines at the maximum doses allowed by OIV when existent or at the maximum dose recommended by the supplier when no OIV limit exists. Their performance was tested in both white and red wines to evaluate if the phenolic composition of the matrix interfered with aflatoxins’ adsorption.

**Figure 1:**
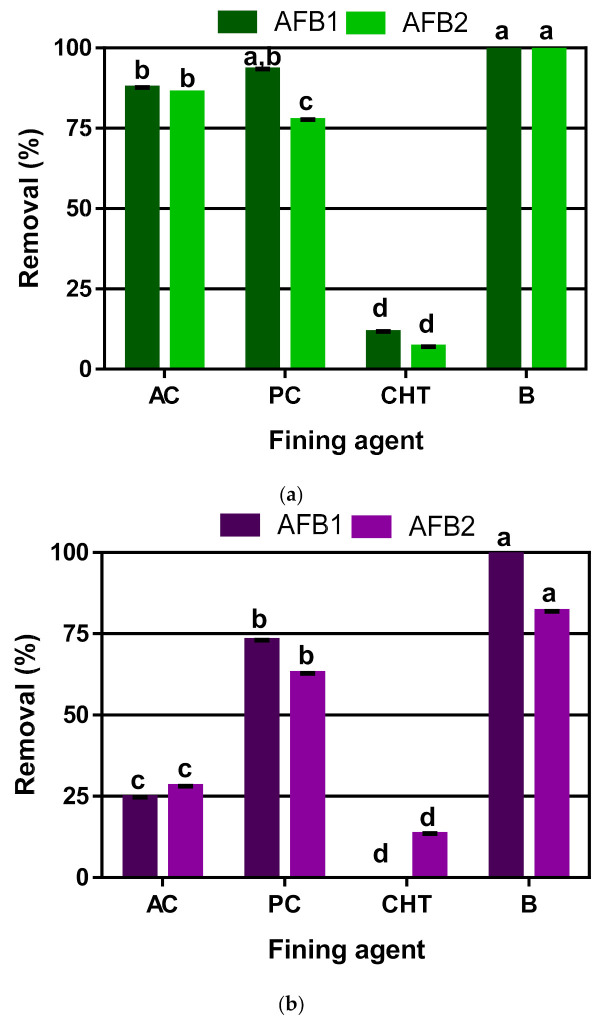
Removal of aflatoxins B1 (AFB1) and B2 (AFB2) in white (**a**) and red wine (**b**) using different fining agents. Means within a column for each wine, followed by the same letter are not significantly different (Tukey *p* < 0.05). AC—Activated Carbon; PC—Potassium Caseinate; CHT—Chitosan; B—Bentonite.

**Figure 2:**
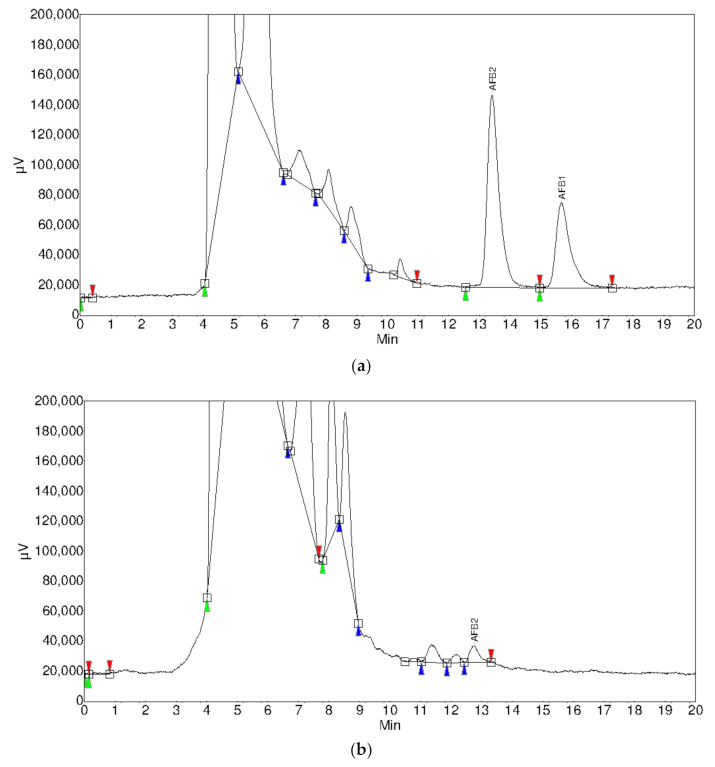
Chromatograms showing the content of aflatoxins B1 and B2 in (**a**) control red wine and (**b**) after treatment with 120 g/hL of bentonite.

The removal percentage of AFB1 and AFB2 in white and red wines are shown in Figure 1. As expected, it was observed a higher efficiency of the fining agents on white wine than on red wine (3.3 times higher for activated carbon, 1.3 times higher for potassium caseinate, 1.4 times higher for chitosan, and only 1.1 times higher for bentonite). Bentonite showed the best performance in both types of wine, with removal percentages of 100% for both aflatoxins in white wine and percentages of 100 and 82% in red wine for AFB1 and AFB2, respectively (Figure 2). The excellent performance of bentonite can be explained by the polarity of AFB1, which enhances its binding or adsorption by the silicate material. Aflatoxin binding to phyllosilicate clay occurs on contact with negligible dissociation of the complex at equilibrium [33]. These authors described that aflatoxin’s *p*-dicarbonyl system was found to be essential for tight binding by phyllosilicate clay, indicating that the molecular mechanism of aflatoxin binding may involve the chelation of metal ions in phyllosilicate clay with the *p*-dicarbonyl moiety of aflatoxin molecule.

The second most efficient fining agent was potassium caseinate with removal percentages in white wine of 93% and 73% for AFB1 and AFB2, respectively; while in red wine, they were 77% and 63%. Activated carbon also showed a good performance in white wine, reaching removal percentages of 86 and 88% for AFB1 and AFB2, respectively. Nevertheless, its performance was much lower in red wine, showing removal percentages of only 25% and 30% for AFB1 and AFB2, respectively. The adsorption of aflatoxin on activated carbon can be explained by the existence of accessible pores in the carbon structure with adequate size for aflatoxin [34]. Additionally, the presence in the matrix of competing substances for the same pores; for example, anthocyanins of red wine, can explain this fining agent’s lower efficiency for aflatoxin removal in red wine. Chitosan was the least efficient fining agent with low removal percentages (<12%) in both types of wine. These results show that bentonite is an excellent solution for removing these aflatoxins in both white and red wines.

### 3.2. Effect of Fining Agents on Wines Chromatic Characteristics

In order to study the impact of applying the different fining agents on wine quality after AFB1 and AFB2 removal, the wine color and chromatic characteristics were measured. Results are presented in Table 2 (for white wine) and Table 3 (for red wine). White wine color (Abs_420 nm_) decreased significantly with the application of all fining agents, except with chitosan. Bentonite and potassium caseinate were the fining agents that resulted in a lower white wine color (Table 2). 

For white wines, a* values did not change significantly after applying the fining agents. On the other hand, L* value was significantly affected by the use of activated carbon. The decrease observed in white wine b* values agree with the decrease observed in wine color (Abs _420 nm_). Only the treatment of white wine with activated carbons resulted in a ΔE* value of 4.8 which is perceptible to the human eye [35]. For the other fining agents, the color differences were below 2. A low brown-yellow color is considered a positive feature in white wine’s sensory quality; therefore, the use of bentonite for the removal of aflatoxins in white wines will not negatively affect their color.

On the other hand, in red wines, a significant decrease in the color intensity was observed with the application of all fining agents, except again with chitosan (Table 3). The higher decrease was observed with bentonite (13.4%). Concerning the chromatic characteristics (CIELab), only the activated carbon and chitosan did not change the red wine L* and a* values significantly. In the wines fined with potassium caseinate and bentonite, the changes observed for the L* values agree with the decrease observed in color intensity, while the changes of a* values are in accordance with the decrease in total phenols, flavonoid phenols, total anthocyanins, colored anthocyanins, and polymeric pigments. The application of potassium caseinate (ΔE* = 4.17) and bentonite (ΔE* = 4.80) in red wines resulted in color differences higher than 1 [36]. 

Although these color differences can be probably detected by the human eye and considered negatively by the consumers, the color intensity of wines only decreased 13.4% with bentonite; this decrease can be acceptable if one considers that the fining agent decreased the contents of AFB1 and AFB2 in 100% and 82%, respectively. It should also be highlighted that the experiments were conducted in the worst scenario situation, thus for lower levels of aflatoxins, they will be necessary lower doses of bentonite, and the effect on wine color will be inferior.

### 3.3. Effect of Fining Agents on Wine Total Phenolic Compounds, Flavonoid, Non-Flavonoid Compounds, and Individual Phenolic Compounds

In order to explain the changes in wines’ chromatic characteristics after applying the fining agents, the total phenolic compounds, flavonoid, non-flavonoid, and individual phenolic compounds were determined and studied by high-performance liquid chromatography with a diode-array detector (HPLC-DAD). In white wines, the activated carbon, potassium caseinate, and bentonite decreased the total phenols by 7%, 1%, and 3%, respectively (Table 2), in line with the observed decreases of b* values (Table 2). For activated carbon, the decrease in total phenols is due to the reduction of flavonoid phenols (4%) and non-flavonoid phenols (15%), while for bentonite, the reduction was only due to flavonoid phenols (3%) (Table 2). The results obtained for the individual phenolic compounds (Table 4) align with that of the total phenols, flavonoid phenols, and non-flavonoid phenols determined colorimetrically. The phenolic acids removed in higher amounts by activated carbon, potassium caseinate, and bentonite were *trans*-caftaric and coutaric acids, the most abundant phenolic acids present in white wine. However, all phenolic acids were decreased with the application of these fining agents (Table 4). Chitosan presented the lowest impact in the white wine phenolic compounds (Table 4).

Generally, the reduction trend registered in white wines for flavonoid, non-flavonoid, and individual phenolic compounds was also observed in red wines, but, in this last case, anthocyanins were the most affected phenolic compounds. Activated carbon (1%), potassium caseinate (3%), and bentonite (6%) decreased the total phenols in line with the observed change in the decrease in a* values of red wines (Table 3). This decrease in total phenols is due to a decrease in total anthocyanins (2%) and non-flavonoid phenols (1%) for activated carbon; total anthocyanins (7%) and non-flavonoid phenols (8%) for potassium caseinate; and total anthocyanins (14%) and non-flavonoid phenols (5%) for bentonite (Table 3). The results obtained for the individual phenolic compounds and monomeric anthocyanins (Table 5 and Table 6, respectively) followed the same reduction trend described for total phenols, flavonoid phenols, non-flavonoid phenols, and total anthocyanins determined colorimetrically. Although all phenolic acids concentration has decreased with the fining agents, gallic acid, *trans*-caftaric acid, and catechin were the most affected by activated carbon, potassium caseinate, and bentonite (Table 5). Therefore, the reduction in red wine chromatic characteristics is due to the removal of anthocyanins and also probably due to the removal of simple phenolic co-pigments like catechin and the ethyl ester of coumaric acid [37] (Table 5).

Nonetheless, it should also be highlighted that they were the most abundant phenolic acids in red wine. For the monomeric anthocyanins, those present in higher abundance were the most removed in absolute amounts, namely malvidin-3-*O*-glucoside, malvidin-3-*O*-acetylglucoside, petunidin-3-*O*-glucoside, and cyanidin-3-*O*-glucoside (Table 6). Chitosan was again the fining agent with the lowest impact in the wine phenolic compounds (Table 5).

Due to the excellent results obtained in aflatoxin B1 and B2 removal from white and red wines by application of bentonite, it would be interesting to reduce the negative effect observed on the red wine color. Therefore, further work will be performed in order to compare the efficiency of different bentonites [natural bentonites (sodium and calcium) and activated bentonites (calcium)] in the removal performance of aflatoxin B1 and B2 and also their effect on the red wine color. In addition, the efficiency of the timing of bentonite application could be explored in order to increase its efficiency and reduce its negative effects, i.e., in the grape must versus final wine.

## 4. Conclusions

The results obtained in this work allow us to conclude that:

- Aflatoxin B1 and B2, two highly toxic mycotoxins, can be eliminated almost entirely from white and red wines with bentonite at 120 g/hL application dose, an already authorized fining agent and dosage in winemaking by the OIV.
- The impact of bentonite in white wine color was residual, while in red wines, a 13% decrease in the color intensity was observed.
- The gain achieved in wine safety and the low impact on wine color, bentonite can be considered an excellent solution for dealing with the aflatoxin safety problem in wines.

## Figures and Tables

**Table 1:** Physicochemical characteristics from the white and the red wines used in the experiments.

Parameters	White Wine	Red Wine
Alcohol content (% *v*/*v*)	10.0	13.0
Specific gravity at 20 °C (g/mL)	0.9915	0.9918
Titratable acidity (g/L tartaric acid)	6.7	5.2
pH	3.00	3.47
Volatile acidity (g/L acetic acid)	0.14	0.40

**Table 2:** Effect of fining agents on phenolic compounds, color (Abs_420 nm_), and chromatic characteristics of white wine.

Parameters	Control	AC	PC	CHT	B
Total phenols (mg/L GA)	154 ± 0 ^a^	143 ± 2 ^c^	152 ± 1 ^b^	154 ± 0 ^a^	150 ± 1 ^b^
Flavonoid phenols (mg/L GA)	106 ± 1 ^a^	102 ± 2 ^c^	106 ± 1 ^b^	105 ± 2 ^a^	103 ± 2 ^a^
Non-flavonoid phenols (mg/L GA)	48 ± 1 ^ab^	41 ± 0 ^b^	45 ± 1 ^b^	48 ± 2 ^a^	47 ± 3 ^a^
Color (Abs_420 nm_)	0.081 ± 0.002 ^a^	0.077 ± 0.001 ^b^	0.067 ± 0.000 ^c^	0.081 ± 0.000 ^a^	0.068 ± 0.001 ^c^
L*	97.3 ± 0.1 ^a^	92.6 ± 1.0 ^b^	98.2 ± 0.1 ^a^	97.4 ± 0.0 ^a^	97.9 ± 0.5 ^a^
a*	−0.18 ± 0.01 ^ab^	−0.24 ± 0.01 ^ab^	−0.30 ± 0.01 ^a^	−0.20 ± 0.01 ^ab^	−0.14 ± 0.07 ^b^
b*	5.21 ± 0.01 ^a^	4.56 ± 0.01 ^ab^	4.30 ± 0.03 ^ab^	5.19 ± 0.11 ^a^	4.07 ± 0.58 ^b^
ΔE*	-	4.76 ± 0.89 ^b^	1.25 ± 0.01 ^a^	0.14 ± 0.08 ^a^	1.35 ± 0.32 ^a^

Values are presented as mean ± standard deviation (n = 2); Means within a line followed by the same letter are not significantly different (Tukey *p* < 0.05). GA—Gallic acid; AC—Activated Carbon; PC—Potassium Caseinate; CHT—Chitosan; B—Bentonite. L* for the lightness from black (0) to white (100), a* from green (−) to red (+), and b* from blue (−) to yellow (+), ΔE*—Color difference in relation to control.

**Table 3:** Effect of fining agents’ application on phenolic compounds and chromatic characteristics of red wine.

Parameters	Control	AC	PC	CHT	B
Total phenols (mg/L GA)	1691 ± 7 ^a^	1669 ± 6 ^b^	1639± 6 ^c^	1691 ±7 ^a^	1585 ±0 ^d^
Flavonoid phenols (mg/L GA)	1421 ± 10 ^a^	1403 ± 8 ^b^	1389 ± 7 ^c^	1428 ± 5 ^a^	1328 ± 3 ^d^
Non-flavonoid phenols (mg/L GA)	270 ± 3 ^a^	266 ± 4 ^a^	249 ± 3 ^c^	262 ± 3 ^b^	256 ± 3 ^b^
Total anthocyanins (mg/L)	343 ± 2 ^a^	336 ± 1 ^b^	318 ± 3 ^c^	333 ± 2 ^b^	296 ± 2 ^d^
Colored anthocyanins (a.u)	4.80 ± 0.11 ^a^	4.44 ± 0.45 ^ab^	4.16 ± 0.04 ^bc^	4.74 ± 0.08 ^a^	3.85 ± 0.05 ^c^
Polymeric pigments (a.u.)	6.05 ± 0.10 ^a^	5.73 ± 0.41 ^a^	5.28 ± 0.04 ^b^	6.02 ± 0.09 ^a^	5.09 ± 0.04 ^b^
Total pigments (a.u.)	16.36 ± 0.71 ^a^	16.11 ± 0.17 ^a^	14.97 ± 0.22 ^b^	16.31 ± 0.06 ^a^	13.91 ± 0.15 ^c^
Color intensity (a.u.)	11.57 ± 0.11 ^a^	11.12 ± 0.21 ^b^	10.18 ± 0.08 ^c^	11.49 ± 0.09 ^ab^	10.02 ± 0.05 ^c^
Hue	0.68 ± 0.01 ^a^	0.71 ± 0.05 ^ab^	0.69 ± 0.01 ^a^	0.68 ± 0.01 ^a^	0.73 ± 0.01 ^b^
L*	76.0 ± 0.1 ^a^	76.4 ± 0.1 ^a^	78.5 ± 0.2 ^b^	76.0 ± 0.1 ^a^	78.4 ± 0.0 ^b^
a*	31.80 ± 0.53 ^a^	31.47 ± 0.01 ^a^	28.53 ± 0.40 ^b^	31.65 ± 0.37 ^a^	27.69 ± 0.39 ^b^
b*	4.10 ± 0.16 ^a^	4.23 ± 0.08 ^a^	3.78 ± 0.03 ^a^	3.88 ± 0.05 ^a^	3.72 ± 0.56 ^a^
ΔE*	-	0.45 ± 0.03 ^a^	4.17 ± 0.02 ^b^	0.30 ± 0.00 ^a^	4.80 ± 0.18 ^c^

Values are presented as mean ± standard deviation (n = 2); Means within a line followed by the same letter are not significantly different (Tukey *p* < 0.05). a.u.—absorbance units; GA—Gallic acid; AC—Activated Carbon; PC—Potassium Caseinate; CHT—Chitosan; B—Bentonite. L* for the lightness from black (0) to white (100), a* from green (−) to red (+), and b* from blue (−) to yellow (+), ΔE*—Color difference in relation to control.

**Table 4:** Effect of fining agents’ application on phenolic acids (mg/L) and flavonoids (mg/L) in white wine.

Phenolic Acids and Flavonoids	Control	AC	PC	CHT	B
Gallic acid	3.00 ± 0.86 ^a^	2.57 ± 0.83 ^a^	1.93 ± 0.11 ^a^	2.22 ± 0.07 ^a^	2.61 ± 0.05 ^a^
Catechin	2.86 ± 0.11 ^a^	2.21 ± 0.61 ^a^	2.39 ± 0.10 ^a^	2.25 ± 0.00 ^a^	2.49 ± 0.02 ^a^
*trans*-Caftaric acid	7.19 ± 0.96 ^a^	5.99± 1.43 ^a^	5.88 ± 1.87 ^a^	7.20 ± 0.95 ^a^	5.55 ± 0.58 ^a^
Coutaric acid	6.22 ± 0.52 ^a^	4.62 ± 0.11 ^b^	5.49 ± 0.57 ^ab^	6.07 ± 0.50 ^a^	4.99 ± 0.39 ^b^
Caffeic acid	0.65 ± 0.02 ^a^	0.56 ± 0.22 ^a^	0.65 ± 0.13 ^a^	0.62 ± 0.01 ^a^	0.48 ± 0.01 ^a^
*p*-Coumaric acid	1.24 ± 0.09 ^a^	0.47 ± 0.31 ^b^	1.25 ± 0.18 ^a^	0.99 ± 0.28 ^ab^	1.21 ± 0.25 ^a^
Ferulic acid	0.55 ± 0.16 ^a^	0.15 ± 0.03 ^b^	0.53 ± 0.09 ^a^	0.56 ± 0.06 ^a^	0.40 ± 0.02 ^ab^
Ethyl ester of caffeic acid	0.94 ± 0.03 ^a^	0.11 ± 0.02 ^d^	0.81 ± 0.02 ^b^	0.89 ± 0.03 ^ab^	0.70 ± 0.03 ^c^
Ethyl ester of coumaric acid	0.36 ± 0.04 ^a^	0.01 ± 0.02 ^b^	0.23 ± 0.13 ^ab^	0.33 ± 0.00 ^a^	0.22 ± 0.00 ^ab^

Values are presented as mean ± standard deviation (n = 2); Means within a line followed by the same letter are not significantly different (Tukey *p* < 0.05). AC—Activated Carbon; PC—Potassium Caseinate; CHT—Chitosan; B—Bentonite.

**Table 5:** Effect of fining agents’ application on phenolic acids (mg/L) and flavonoids (mg/L) in red wine.

Phenolic Acids and Flavonoids	Control	AC	PC	CHT	B
Gallic acid	18.99 ± 1.59 ^a^	17.62 ± 1.77 ^a^	16.72 ± 0.11 ^a^	16.16 ± 1.77 ^a^	14.11 ± 3.66 ^a^
Catechin	18.35 ± 4.04 ^a^	15.33 ± 3.77 ^a^	13.32 ± 0.22 ^a^	15.95 ± 4.49 ^a^	13.88 ± 0.59 ^a^
*trans*-Caftaric acid	29.39 ± 1.33 ^a^	25.27 ± 0.64 ^b^	29.72 ± 0.24 ^a^	29.08 ± 0.43 ^a^	29.37 ± 0.75 ^a^
Coutaric acid isomer	0.48 ± 0.00 ^a^	0.35 ± 0.05 ^b^	0.48 ± 0.00 ^a^	0.42 ± 0.09 ^a^	0.49 ± 0.01 ^a^
Coutaric acid	10.54 ± 0.36 ^ab^	10.03 ± 0.04 ^b^	10.53 ± 0.09 ^ab^	10.82 ± 0.54 ^a^	10.57 ± 0.03 ^ab^
Caffeic acid	2.84 ± 0.04 ^a^	1.07 ± 0.81 ^b^	2.72 ± 0.03 ^a^	2.83 ± 0.00 ^a^	2.85 ± 0.00 ^a^
*p*-Coumaric acid	2.26 ± 0.31 ^ab^	2.11 ± 0.08 ^b^	2.36 ± 0.09 ^ab^	2.59 ± 0.12 ^b^	2.46 ± 0.04 ^ab^
Ferulic acid	0.90 ± 0.01 ^a^	0.60 ± 0.13 ^ab^	0.36 ± 0.00 ^b^	0.51 ± 0.10 ^b^	0.49 ± 0.15 ^b^
Ethyl ester of caffeic acid	0.55 ± 0.01 ^a^	0.25 ± 0.21 ^b^	0.50 ± 0.01 ^a^	0.55 ± 0.02 ^a^	0.53 ± 0.01 ^a^
Ethyl ester of coumaric acid	2.70 ± 0.52 ^a^	1.74 ± 0.03 ^b^	1.70 ± 0.07 ^b^	2.17 ± 0.02 ^ab^	1.43 ± 0.00 ^b^

Values are presented as mean ± standard deviation (n = 2); Means within a line followed by the same letter are not significantly different (Tukey *p* < 0.05). AC—Activated Carbon; PC—Potassium Caseinate; CHT—Chitosan; B—Bentonite.

**Table 6:** Effect of fining agents’ application on monomeric anthocyanins (mg/L) in red wine.

Anthocyanins	Control	AC	PC	CHT	B
D-3-G	2.54 ± 0.04 ^a^	2.26 ± 0.25 ^a^	2.65 ± 0.10 ^a^	2.30 ± 0.25 ^a^	2.56 ± 0.12 ^a^
C-3-G	10.82 ± 0.48 ^a^	4.35 ± 0.09 ^d^	8.81 ± 0.08 ^b^	10.39 ± 0.02 ^a^	5.83 ± 0.06 ^c^
Pet-3-G	16.63 ± 0.37 ^a^	12.61 ± 0.55 ^c^	14.13 ± 0.19 ^b^	16.54 ± 0.35 ^a^	10.11 ± 0.09 ^d^
Peo-3-G	19.03 ± 0.52 ^a^	7.89 ± 1.11 ^c^	17.10 ± 0.22 ^a^	18.64 ± 0.42 ^a^	12.93 ± 0.79 ^b^
M-3-G	90.40 ± 2.57 ^a^	78.89 ± 1.43 ^b^	78.64 ± 0.69 ^b^	90.93 ± 0.71 ^a^	56.78 ± 1.49 ^v^
D-3-A	5.90 ± 0.05 ^a^	5.14 ± 0.14 ^b^	4.92 ± 0.07 ^b^	6.08 ± 0.02 ^a^	3.13 ± 0.05 ^c^
C-3-A	0.74 ± 0.03 ^a^	0.66 ± 0.05 ^ab^	0.59 ± 0.01 ^ab^	0.70 ± 0.19 ^a^	0.47 ± 0.03 ^b^
Pet-3-A	1.66 ± 0.23 ^a^	n.d.	0.36 ± 0.13 ^c^	1.61 ± 0.07 ^a^	1.00 ± 0.05 ^b^
Peo-3-A	2.04 ± 0.16 ^a^	1.30 ± 0.14 ^b^	1.57 ± 0.46 ^ab^	1.99 ± 0.28 ^a^	1.19 ± 0.14 ^b^
M-3-A	12.42 ± 3.77 ^a^	10.32 ± 2.05 ^ab^	8.65 ± 0.15 ^ab^	9.66 ± 0.22 ^ab^	6.13 ± 0.02 ^b^
D-3-C	0.78 ± 0.18 ^a^	0.25 ± 0.01 ^b^	0.40 ± 0.03 ^b^	0.57 ± 0.05 ^ab^	0.48 ± 0.04 ^ab^
C-3-C	0.71 ± 0.02 ^a^	0.58 ± 0.45 ^a^	0.74 ± 0.01 ^a^	0.35 ± 0.08 ^a^	0.42 ± 0.01 ^a^
Pet-3-C	1.76 ± 0.16 ^ab^	1.95 ± 0.01 ^a^	1.50 ± 0.13 ^b^	1.80 ± 0.06 ^ab^	0.98 ± 0.04 ^c^
M-3-C	15.28 ± 0.61 ^a^	11.11 ± 0.12 ^b^	11.53 ± 0.29 ^b^	14.48 ± 0.40 ^a^	9.31 ± 0.07 ^c^

Values are presented as mean ± standard deviation (n = 2); Delphinidin-3--*O*-glucoside (D-3-G), Cyanidin-3-*O*-glucoside (C-3-G), Petunidin-3--*O*-glucoside (Pet-3-G), Peonidin-3--*O*-glucoside (Peo-3-G), Malvidin-3--*O*-glucoside (M-3-G), Delphinidin-3--*O*-acetylglucoside (D-3-A), Cyanidin-3--*O*-acetylglucoside (C-3-A), Petunidin-3--*O*-acetylglucoside (Pet-3-A), Peonidin-3--*O*-acetylglucoside (Peo-3-A), Malvidin-3--*O*-acetylglucoside (M-3-A), Cyanidin-3--*O*-coumaroylglucoside (C-3-C), Malvidin-3--*O*-coumaroylglucoside (M-3-C), Means within a line followed by the same letter are not significantly different (Tukey *p* < 0.05). n.d. not detected; AC—Activated Carbon; PC—Potassium Caseinate; CHT—Chitosan; B—Bentonite.

## References

[1] Deiner U.L., Cole R.J., Sanders T.H., Payne G.A., Lee L.S., Kich M.A. (1987). Epidemiology of aflatoxin formation by *Aspergillus flavus*. Annu. Rev. Phytopathol..

[2] Kutrzman C.P., Horn B.W., Hesseltine C.W. (1987). *Aspergillus nominus*, a new aflatoxin-producing species related to *Aspergillus flavus* and *Aspergillus tamarii*. Antonie Van Leeuwenhoek.

[3] Williams J.H., Phillips T.D., Jolly P.E., Stiles J.K., Jolly C.M., Aggarwal D. (2004). Human aflatoxicosis in developing countries: A review of toxicology, exposure, potential health consequences and interventions. Am. J. Clin. Nutr..

[4] Moss M. (1998). Recent studies of mycotoxins. J. Appl. Microbiol..

[5] JECFA (1999). Evaluation of Certain Food Additives and Contaminants. https://apps.who.int/iris/bitstream/handle/10665/42142.

[6] Busby W.F., Wogan G.N. (1984). Aflatoxin. Chemical Carcinogens.

[7] Hamid A.S., Tesfamariam I.G., Zhang Y., Zhang Z.G. (2013). Aflatoxin B1-induced hepatocellular carcinoma in developing countries: Geographical distribution, mechanism of action and prevention. Oncol. Lett..

[8] Sweeney M.J., Dobson A.D.W. (1998). Mycotoxin production by *Aspergillus*, *Fusarium* and *Penicillium* species. Int. J. Food Microbiol..

[9] Kensler T.W., Roebuck B.D., Wogan G.N., Groopman J.D. (2011). Aflatoxin: A 50-year odyssey of mechanistic and translational toxicology. Toxicol. Sci..

[10] IARC (1993). IARC Working Group on the Evaluation of Carcinogenic Risk to Humans. Some Naturally Occurring Substances: Food Items and Constituents, Heterocyclic Aromatic Amines and Mycotoxins. Lyon (FR): International Agency for Research on Cancer. IARC Monographs on the Evaluation of Carcinogenic Risks to Humans. https://www.ncbi.nlm.nih.gov/books/NBK513568/.

[11] Pérez-Ortega P., Gilbert-López B., García-Reyes J.F., Ramos-Martos N., Molina-Díaz A. (2012). Generic sample treatment method for simultaneous determination of multiclass pesticides and mycotoxins in wines by liquid chromatography–mass spectrometry. J. Chromatogr. A.

[12] Di Stefano V., Pitonzo R., Avellone G., Di Fiore A., Monte L., Zofia A., Ogorka T. (2015). Determination of aflatoxins and ochratoxins in Sicilian sweet wines by High-Performance Liquid Chromatography with fluorometric detection and immunoaffinity. Food Anal. Methods.

[13] Di Stefano V., Avellone G., Pitonzo R., Capocchiano V.G., Mazza A., Cicero N., Dugo G. (2015). Natural co-occurrence of ochratoxin A, ochratoxin B and aflatoxins in Sicilian red wines. Food Addit. Contam. Part A.

[14] Nistor A.-M., Cotan S.-D., Cotea V.V., Niculaua N. Analysis of aflotoxins in rustically wines from eastern Romania using the direct real time method (DART). Proceedings of the 41st World Congress of Vine and Wine. BIO Web of Conferences.

[15] El Khoury A., Rizk T., Lteif R., Azouri H., Delia M.-L., Lebrihi A. (2008). Fungal contamination and Aflatoxin B1 and Ochratoxin A in Lebanese wine–grapes and musts. Food Chem. Toxicol..

[16] Fredj S.M.B., Chebil S., Mliki A. (2009). Isolation and characterization of ochratoxin A and aflatoxin B1 producing fungi infecting grapevines cultivated in Tunisia. Afr. J. Microbiol. Res..

[17] Paterson R.R.M., Lima N. (2010). How will climate change affect mycotoxins in food?. Food Res. Int..

[18] Paterson R.R.M., Lima N. (2017). Thermophilic fungi to dominate aflatoxigenic/mycotoxigenic fungi on food under global warming. Int. J. Environ. Res..

[19] ICMSF International Commission on Microbiological Specifications for Foods (1996). Toxigenic fungi: Aspergillus. Microorganisms in Foods 5: Characteristics of Microbial Pathogens.

[20] OIV 2019 Statistical Report on World Vitiviniculture. http://oiv.int/public/medias/6782/oiv-2019-statistical-report-on-world-vitiviniculture.pdf.

[21] Fernandez-Mar M.I., Mateos R., García-Parrilla M.C., Puertas B., Cantos-Villar E. (2012). Bioactive compounds in wine: Resveratrol, hydroxytyrosol and melatonin: A review. Food Chem..

[22] European Union (2006). Commission Regulation (EC) No. 1881/2006, of 19 December setting maximum levels for certain contaminants in food stuffs. Off. J. Eur. Union.

[23] European Union (2010). Commission Regulation (EU) 165/2010 amending Regulation (EC) No. 1881/2006 setting maximum levels for certain contaminants in foodstuffs as regards aflatoxins. Off. J. Eur. Union.

[24] OIV International Code of Oenological Practices (2019). International Organisation of Vine and Wine. http://www.oiv.int/fr/normes-et-documents-techniques.

[25] Kramling T., Singleton V.L. (1969). An estimate of the nonflavonoid phenols in wines. Am. J. Enol. Vitic.

[26] Ribéreau-Gayon P., Peynaud E., Sudraud P. (1982). Traité d’Œnologie. Science et Techniques du Vin.

[27] OIV (2015). Organisation International de la Vigne et du Vin Récueil de Méthodes Internationales d’Analyse des Vins et des Moûts. http://www.oiv.org/fr/normes-et-documents-techniques/methodes-danalyse.

[28] Ribéreau-Gayon P., Stronestreet E. (1965). Le dosage des anthocyanes dans le vin rouge. Bull. Soc. Chim. Fr..

[29] Somers T.C., Evans M.E. (1977). Spectral evaluation of young red wines: Anthocyanin equilibria, total phenolics, free and molecular O2, “Chemical age”. J. Sci. Food Agric..

[30] Filipe-Ribeiro L., Milheiro J., Matos C.C., Cosme F., Nunes F.M. (2017). Reduction of 4-ethylphenol and 4-ethylguaiacol in red wine by activated carbons with different physicochemical characteristics: Impact on wine quality. Food Chem..

[31] Filipe-Ribeiro L., Milheiro J., Matos C.C., Cosme F., Nunes F.M. (2017). Data on changes in red wine phenolic compounds, headspace aroma compounds and sensory profile after treatment of red wines with activated carbons with different physicochemical characteristics. Data Brief.

[32] Guise R., Filipe-Ribeiro L., Nascimento D., Bessa O., Nunes F.M., Cosme F. (2014). Comparison between different types of carboxylmethylcellulose and other oenological additives used for white wine tartaric stabilization. Food Chem..

[33] Phillips T., Sarr A., Grant P. (1995). Selective chemisorption and detoxification of aflatoxins by phyllosilicate clay. Nat. Toxins.

[34] Moreno-Castilla C. (2004). Adsorption of organic molecules from aqueous solutions on carbon materials. Carbon.

[35] Spagna G., Barbagallo R.N., Pifferi P.G. (2000). Fining treatments of white wines by means of polymeric adjuvants for their stabilization against browning. J. Sci. Food Agric..

[36] Gonnet J.F. (1998). Colour effects of co-pigmention of anthocyanins revisited-1. A colorimetric definition using the CIElab scale. Food Chem..

[37] Trouillas P., Sancho-García J.C., De Freitas V., Gierschner J., Otyepka M., Dangles O. (2016). Stabilizing and Modulating Color by Copigmentation: Insights from Theory and Experiment. Chem. Rev..

